# The Virasoro fusion kernel and Ruijsenaars’ hypergeometric function

**DOI:** 10.1007/s11005-020-01351-4

**Published:** 2021-01-09

**Authors:** Julien Roussillon

**Affiliations:** Department of Mathematics, KTH Royal Institute of Technology, 100 44 Stockholm, Sweden

**Keywords:** 2d conformal field theory, Integrability, 81T40, 81Q80

## Abstract

We show that the Virasoro fusion kernel is equal to Ruijsenaars’ hypergeometric function up to normalization. More precisely, we prove that the Virasoro fusion kernel is a joint eigenfunction of four difference operators. We find a renormalized version of this kernel for which the four difference operators are mapped to four versions of the quantum relativistic hyperbolic Calogero–Moser Hamiltonian tied with the root system $BC_1$. We consequently prove that the renormalized Virasoro fusion kernel and the corresponding quantum eigenfunction, the (renormalized) Ruijsenaars hypergeometric function, are equal.

## Introduction

Two-dimensional conformal field theories (CFTs) have been intensively studied since the seminal work of Belavin et al. [3]. In addition to their broad range of physical applications in, for example, condensed matter theory and string theory, they possess rich underlying mathematical structures.

The infinite-dimensional symmetry algebra of 2D CFTs determines the structure of their correlation functions. The operator product expansion makes it possible to decompose *N*-point correlators on the Riemann sphere into combinations of three-point structure constants and universal quantities called *Virasoro conformal blocks*. In particular, there are three ways to decompose the four-point correlation function. The universal parts of each decomposition are called *s*-, *t*- and *u*-channel conformal blocks. Since each decomposition must lead to the same four-point function, the three kinds of conformal blocks are related by duality transformations.

In particular, the *s*- and *t*-channel conformal blocks are related by an integral transform called fusion transformation. Such a transformation was conjectured in [21] and established in [36]. The corresponding kernel, the *Virasoro fusion kernel*, was constructed by Ponsot and Teschner [21, 22] and revisited in [38] as *b-6j symbols* for the modular double of $\mathcal {U}_q(sl_2(\mathbb {R}))$. This terminology comes from the fact that *b*, which is related to the central charge of the Virasoro algebra by $c=1+6(b+b^{-1})^2$, is associated with two unimodular parameters $q=e^{i\pi b^2}$ and $\tilde{q}=e^{i\pi b^{-2}}$. The Virasoro fusion kernel also appears in quantum Teichmüller theory [20] and in 3d supersymmetric gauge theories [38, 39], and recent physical applications were found in [6, 7, 12].

The Virasoro fusion kernel is denoted $F\left( b,{\varvec{\theta }},\sigma _s,\sigma _t \right)$. Here, ${\varvec{\theta }}$ is a set of four external conformal dimensions associated with the fields entering the four-point correlation function, while $\sigma _s$ and $\sigma _t$ are two internal conformal dimensions associated with the *s*- and *t*-channel decompositions of the four-point function.

The Ponsot–Teschner formula [21] for *F* is recalled in (4.1). It is given by a contour integral whose integrand involves a special function $s_b(z)$. The $s_b$-function appears in several different contexts. It was introduced under the names of “Quantum Dilogarithm function,” “Hyperbolic Gamma function” and “Quantum Exponential function” in [9, 25, 41], respectively.^1^ In particular, *F* is proportional to a *hyperbolic Barnes integral* which is a hyperbolic generalization of the Barnes representation for the Gauss hypergeometric function. Such an integral was constructed in [5] as a degeneration limit of the hyperbolic hypergeometric function, and it also arises as a limit of Spiridonov’s elliptic hypergeometric *V*-function [32, 38]. Finally, the Virasoro fusion kernel is a joint eigenfunction of four difference operators [17, 21].

On the other hand, Calogero–Moser (CM) models are systems of particles living on the real line or the circle and interacting with a rational, trigonometric/hyperbolic or elliptic potential. Their relativistic deformations were found by Ruijsenaars and Schneider at the classical level [31]. Quantum relativistic CM systems tied with the root system $A_{N-1}$ were found by Ruijsenaars [30], and their $BC_N$ generalization by van Diejen [40]. Therefore, we will refer to quantum relativistic $BC_N$ CM systems as quantum Ruijsenaars–van Diejen (RvD) systems. In this article, only the case $N=1$ of the latter system will be considered.

The rank *N* quantum trigonometric RvD system is solved by the Koornwinder *N*-variable polynomials [13]. However, in the hyperbolic case the eigenfunctions are non-polynomial and are only known in the rank one. The corresponding eigenfunction, the *Ruijsenaars hypergeometric function*, was introduced in [24] and studied in greater detail in [26–28]. This function is denoted $R(a_-,a_+,\varvec{\gamma },v,\hat{v})$; here, $a_-$ and $a_+$ are associated with two unimodular quantum deformation parameters $q=e^{i\pi a_-/a_+}$ and $\tilde{q}=e^{i \pi a_+/a_-}$, while ${\varvec{\gamma }}$ is a set of four external couplings constants. Finally, *v* and $\hat{v}$ are viewed as geometric and spectral variables, respectively.

Of particular importance is the renormalized *R*-function, denoted $R_\text {ren}(a_-,a_+,\varvec{\gamma },v,\hat{v})$, which was defined in [26]. The definition (2.3) and the properties of the function $R_\text {ren}$ resemble those of the Virasoro fusion kernel. First of all, $R_\text {ren}$ is defined as a contour integral whose integrand involves the hyperbolic gamma function $G(a_-,a_+,z)$. The functions *G* and $s_b$ are simply related by

$$\begin{aligned} s_b(z) = G(b,b^{-1},z). \end{aligned}$$

$R_\text {ren}$ is also proportional to a hyperbolic Barnes integral [5]. Moreover, it is a joint eigenfunction of four difference operators which are four versions of the rank one quantum hyperbolic RvD Hamiltonian. Let us finally mention that the function $R_\text {ren}$ was related to the modular double of $\mathcal {U}_q(sl_2(\mathbb {R}))$ in [4].

The discussion above suggests that the Virasoro fusion kernel and the Ruijsenaars hypergeometric function are closely related. The aim of this article is to show that the two functions are equal up to normalization (see Theorem 1). The identification procedure can be summarized as follows. We prove in Propositions 4.3 and 4.4 that the Virasoro fusion kernel is a joint eigenfunction of four difference operators. We provide the Ruijsenaars/CFT parameters identification in Sect. 5.1. We define in (5.14) a renormalized version $F_\text {ren}$ of *F*, and we show in Proposition 5.3 that both $R_\text {ren}$ and $F_\text {ren}$ satisfy the same four difference equations. Therefore, the two functions are proportional. We finally show in Theorem 1 that they are actually equal: The proof is rather simple and follows from the identity (B.4) satisfied by the hyperbolic Barnes integral.

### Organization of the paper

The Ruijsenaars hypergeometric function and various of its properties are recalled in Sect. 2. Section 3 reviews some properties of the four-point Virasoro conformal blocks and introduces the Virasoro fusion kernel in this context. In Sect. 4, we study various symmetry and eigenfunction properties of *F*. Finally, our main result—the identification of *F* with $R_\text {ren}$—is presented in Sect. 5.

## Ruijsenaars’ hypergeometric function

This section is a brief overview of the (renormalized) Ruijsenaars hypergeometric function following [26]. We recall several of its properties which we will need to relate it to the Virasoro fusion kernel.

### Definition

Define two sets of external couplings constants ${\varvec{\gamma }}$ and $\hat{\varvec{\gamma }}$ by

2.1 $$\begin{aligned} {\varvec{\gamma }}=\begin{pmatrix}\gamma _0\\ \gamma _1 \\ \gamma _2\\ \gamma _3\end{pmatrix}, \qquad \hat{\varvec{\gamma }}=\begin{pmatrix}\hat{\gamma }_0\\ \hat{\gamma }_1 \\ \hat{\gamma }_2\\ \hat{\gamma }_3\end{pmatrix}= J {\varvec{\gamma }}, \end{aligned}$$

where *J* is a matrix satisfying $J^2=\mathcal {I}_4$ and is defined by

2.2 $$\begin{aligned} J=\frac{1}{2} \begin{pmatrix}1 &{} 1 &{} 1 &{} 1 \\ 1 &{} 1 &{} -1 &{}-1\\ 1&{}-1&{}1&{}-1\\ 1&{}-1&{}-1&{}1 \end{pmatrix}. \end{aligned}$$

The renormalized *R*-function denoted $R_\text {ren}(a_-,a_+,\varvec{\gamma },v,\hat{v})$ is given by a contour integral whose integrand involves the hyperbolic gamma function $G(a_-,a_+,z)$. The definition of *G* and its properties can be found in Appendix A. Its dependence on $a_-$ and $a_+$ will be omitted for simplicity. Following [26], the function $R_\text {ren}$ is defined for $(a_-,a_+,\varvec{\gamma }) \in \text {RHP}^2 \times \mathbb {C}^4$, where RHP denotes the open right-half plane, such that

2.3 $$\begin{aligned}&R_{\text {ren}}(a_-,a_+,\varvec{\gamma },v,\hat{v})=\frac{1}{\sqrt{a_+ a_-}} \prod _{\epsilon =\pm } \frac{G(\epsilon v-i \gamma _0)}{G(\epsilon \hat{v}+i \hat{\gamma }_0)}\nonumber \\&\quad \displaystyle \int _{\mathsf {R}} dz \frac{\prod _{\epsilon =\pm }G(z + \epsilon v+i \gamma _0)G(z + \epsilon \hat{v}+i \hat{\gamma }_0)}{G(z+i a)\prod _{j=1}^3 G(z+i \gamma _0+i \gamma _j+i a)}, \end{aligned}$$

and where $a=(a_++a_-)/2$. The function *G* has sequences of poles and zeros given in (A.4) and (A.5), respectively. Therefore, for instance, when $a_+,a_->0$ the integrand in (2.3) has eight semi-infinite lines of poles, four of them increasing and the other ones decreasing. The integration contour $\mathsf {R}$ runs from $-\infty$ to $+\infty$ separating the increasing and decreasing sequences of poles. Moreover, for fixed values of $a_-,a_+$ and $\varvec{\gamma }$, the function $R_\text {ren}$ is meromorphic in *v* and $\hat{v}$.

Finally, it was shown in [5, Proposition 4.20] that the function $R_\text {ren}$ is proportional to a hyperbolic Barnes integral $\mathcal {B}_h(a_-,a_+,\varvec{u})$ whose definition is recalled in Appendix B. $\mathcal {B}_h(a_-,a_+,\varvec{u})$ is one possible degeneration of the hyperbolic hypergeometric function, and other types of degenerations led the authors of [5] to find other representations for $R_\text {ren}$.

### Symmetry properties

The function $R_\text {ren}$ possesses various discrete symmetries which follow from the representation (2.3). In view of the property (A.6) of the hyperbolic gamma function, $R_\text {ren}$ is scale invariant:

2.4 $$\begin{aligned} R_{\text {ren}}(\lambda a_-,\lambda a_+,\lambda \varvec{\gamma },\lambda v,\lambda \hat{v}) = R_{\text {ren}}(a_-,a_+,\varvec{\gamma },v,\hat{v}), \qquad \lambda > 0. \end{aligned}$$

Moreover, because of the fact that $G(a_-,a_+,z)=G(a_+,a_-,z)$, the following identity holds:

2.5 $$\begin{aligned} R_{\text {ren}}(a_-,a_+,\varvec{\gamma },v,\hat{v})=R_{\text {ren}}(a_+,a_-,\varvec{\gamma },v,\hat{v}) \qquad \text {(modular symmetry).} \end{aligned}$$

It can also be verified that $R_\text {ren}$ satisfies

2.6 $$\begin{aligned} R_{\text {ren}}(a_-,a_+,\varvec{\gamma },v,\hat{v})=R_{\text {ren}}(a_-,a_+,\hat{\varvec{\gamma }},\hat{v},v) \qquad \text {(self-duality).} \end{aligned}$$

Finally, the function $R_\text {ren}$ is even in $v,\hat{v}$ and is symmetric under permutations of $\gamma _1,\gamma _2,\gamma _3$. It was shown in [27, Theorem 1.1] that a similarity transformed version of $R_\text {ren}$ has an extended $D_4$-symmetry in $\varvec{\gamma }$. More precisely, define the function $\mathcal {E}_\text {ren}$ by

2.7 $$\begin{aligned} \mathcal {E}_\text {ren}(a_-,a_+,\varvec{\gamma },v,\hat{v}) = \frac{R_{\text {ren}}(a_-,a_+,\varvec{\gamma },v,\hat{v})}{\delta (a_-,a_+,\varvec{\gamma },v)\delta (a_-,a_+,\hat{\varvec{\gamma }},\hat{v})}, \end{aligned}$$

where the function $\delta$ is given by

2.8 $$\begin{aligned} \delta (a_-,a_+,\varvec{\gamma },v) = \frac{\prod _{\mu =0}^3 G(v-i\gamma _\mu )}{G(2v+ia)}. \end{aligned}$$

The function $\mathcal {E}_\text {ren}$ is invariant under all permutations of $\gamma _0,\gamma _1,\gamma _2,\gamma _3$ and under flipping the sign of any pair of $\gamma _\mu$’s. The resulting invariance group is the Weyl group of the Lie algebra $D_4$.

### Eigenfunction properties

Define a translation operator $e^{\pm i a_- \partial _x}$ which formally acts on meromorphic functions *f*(*x*) as $e^{\pm i a_- \partial _x} f(x)=f(x\pm i a_-)$. The quantum relativistic Calogero–Moser Hamiltonian tied with the root system $BC_1$ and with a hyperbolic interaction was defined in [40]. As mentioned in the introduction, it will be referred to as the Ruijsenaars–van Diejen (RvD) Hamiltonian. It is a difference operator of the form

2.9 $$\begin{aligned} H_{RvD}(a_-,a_+,\varvec{\gamma },x)=C(a_-,a_+,\varvec{\gamma },x)e^{-i a_- \partial _x}+C(a_-,a_+,\varvec{\gamma },-x)e^{i a_- \partial _x} + V(a_-,a_+,\varvec{\gamma },x), \end{aligned}$$

where

2.10 $$\begin{aligned} C(a_-,a_+,\varvec{\gamma },x)= -\frac{4\prod _{k=0}^3{\text {cosh}}{\left( \frac{\pi }{a_+}(x-i\gamma _k-\frac{i a_-}{2})\right) }}{{\text {sinh}}{\left( \frac{2\pi x}{a_+}\right) }{\text {sinh}}{\left( \frac{2\pi }{a_+}(x-\frac{i a_-}{2})\right) }}, \end{aligned}$$

and where the potential *V* is defined by

2.11 $$\begin{aligned} V(a_-,a_+,\varvec{\gamma },x)=-C(a_-,a_+,\varvec{\gamma },x)-C(a_-,a_+,\varvec{\gamma },-x)-2{\text {cos}}{\left( \frac{\pi }{a_+}\left( \sum _{\mu =0}^3 \gamma _\mu +a_-\right) \right) }. \end{aligned}$$

The function $R_\text {ren}$ is a joint eigenfunction of four versions of this Hamiltonian [26, Theorem 3.1]. More precisely, for $(a_-,a_+,\varvec{\gamma },v,\hat{v})\in \text {RHP}^2\times \mathbb {C}^6$ the following eigenvalue equations hold:

2.12a $$\begin{aligned}&H_{RvD}(a_-,a_+,\varvec{\gamma },v)R_{\text {ren}}(a_-,a_+,\varvec{\gamma },v,\hat{v})=2{\text {cosh}}{\left( \tfrac{2\pi \hat{v}}{a_+}\right) }R_{\text {ren}}(a_-,a_+,\varvec{\gamma },v,\hat{v}), \end{aligned}$$

2.12b $$\begin{aligned}&H_{RvD}(a_+,a_-,\varvec{\gamma },v)R_{\text {ren}}(a_-,a_+,\varvec{\gamma },v,\hat{v})=2{\text {cosh}}{\left( \tfrac{2\pi \hat{v}}{a_-}\right) }R_{\text {ren}}(a_-,a_+,\varvec{\gamma },v,\hat{v}), \end{aligned}$$

2.12c $$\begin{aligned}&H_{RvD}(a_-,a_+,\hat{\varvec{\gamma }},\hat{v})R_{\text {ren}}(a_-,a_+,\varvec{\gamma },v,\hat{v})=2{\text {cosh}}{\left( \tfrac{2\pi v}{a_+}\right) }R_{\text {ren}}(a_-,a_+,\varvec{\gamma },v,\hat{v}), \end{aligned}$$

2.12d $$\begin{aligned}&H_{RvD}(a_+,a_-,\hat{\varvec{\gamma }},\hat{v})R_{\text {ren}}(a_-,a_+,\varvec{\gamma },v,\hat{v})=2{\text {cosh}}{\left( \tfrac{2\pi v}{a_-}\right) }R_{\text {ren}}(a_-,a_+,\varvec{\gamma },v,\hat{v}). \end{aligned}$$

It can be noted that (2.12a) implies (2.12b), (2.12c) and (2.12d) thanks to the properties (2.5) and (2.6) of $R_\text {ren}$.

## Four-point Virasoro conformal blocks

In this section, we briefly review the four-point Virasoro conformal blocks and recall how the Virasoro fusion kernel arises in this context. A more complete overview of the subject can be found in [23, 35, 36].

### Highest-weight representations of the Virasoro algebra

The Virasoro algebra is the symmetry algebra of two-dimensional conformal field theories. It has generators $L_n$, $n\in \mathbb {Z}$ and relations

3.1 $$\begin{aligned}{}[L_n,L_m]=(n-m)L_{n+m} + \frac{c}{12}n(n^2-1)\delta _{n+m,0},\end{aligned}$$

where *c* is a central element in the algebra called central charge. Highest-weight representations $\mathcal {V}_\theta$ of the Virasoro algebra are generated from vectors $|\theta>$ which satisfy

3.2 $$\begin{aligned} L_0|\theta> = \Delta (\theta ) |\theta>,\qquad L_n|\theta> = 0, \quad n>0, \end{aligned}$$

where a Liouville-type parameterization of $\Delta$ and *c* is used:

3.3 $$\begin{aligned} \Delta (x)=\tfrac{Q^2}{4}+x^2, \qquad c=1+6Q^2,\qquad Q=b+b^{-1}. \end{aligned}$$

The representations $\mathcal {V}_\theta$ decompose as follows:

3.4 $$\begin{aligned} \mathcal {V}_\theta \simeq \bigoplus _{n\in \mathbb {Z} \ge 0} \mathcal {V}_\theta ^{(n)}, \end{aligned}$$

where $L_0 v = (\Delta (\theta )+n) v$ for all $v \in \mathcal {V}_\theta ^{(n)}$. A chiral vertex operator $V^{\Delta (\theta _0)}_{\Delta (\theta _2),\Delta (\theta _1)}(z)$ is a map $V^{\Delta (\theta _0)}_{\Delta (\theta _2),\Delta (\theta _1)} : \mathcal {V}_{\theta _1}\rightarrow \mathcal {V}_{\theta _2}$. It is defined by the following commutation relations:

3.5 $$\begin{aligned}{}[L_n,V^{\Delta (\theta _0)}_{\Delta (\theta _2),\Delta (\theta _1)}(z)]=z^n \left( z \partial _z+\Delta (\theta _0)(n+1)\right) V^{\Delta (\theta _0)}_{\Delta (\theta _2),\Delta (\theta _1)}(z). \end{aligned}$$

In particular, it admits the following formal series expansion:

3.6 $$\begin{aligned} V^{\Delta (\theta _0)}_{\Delta (\theta _2),\Delta (\theta _1)}(z)|\theta _1 >=z^{\Delta (\theta _2)-\Delta (\theta _1)-\Delta (\theta _0)} \sum _{n\in \mathbb {Z}_{\ge 0}} v_n z^n, \end{aligned}$$

where $v_n \in \mathcal {V}_{\theta _2}$ and $v_0=| \theta _2>$. We emphasize that the expansion (3.6) can be renormalized by an arbitrary factor $N(\theta _2,\theta _0,\theta _1)$. Such a factor will be introduced in Sect. 5.

### Four-point Virasoro conformal blocks

We now define a set of external conformal dimensions $\varvec{\theta }$ by

3.7 $$\begin{aligned} \varvec{\theta }=\begin{pmatrix}\theta _0\\ \theta _t \\ \theta _1 \\ \theta _\infty \end{pmatrix}. \end{aligned}$$

The four-point Virasoro conformal block is defined by the expectation value of a composition of two chiral vertex operators as follows:

3.8 $$\begin{aligned} \mathcal F\left( b,\varvec{\theta },\sigma _s;z \right) = \left\langle \theta _\infty \right| V_{\Delta (\theta _\infty ),\Delta (\sigma _s)}^{\Delta (\theta _1)}\left( 1\right) V_{\Delta (\sigma _s),\Delta (\theta _0)}^{\Delta (\theta _t)}\left( z \right) |\theta _0\rangle . \end{aligned}$$

The parameters $\varvec{\theta }$ and $\sigma _s$ will be referred to as external and internal momenta. The block $\mathcal F$ admits a series expansion in *z* which can be computed recursively using (3.1), (3.5) and (3.6):

3.9 $$\begin{aligned} \mathcal F\left( b,\varvec{\theta },\sigma _s;z \right) = z^{\Delta (\sigma _s)-\Delta (\theta _0)-\Delta (\theta _t)}\left( 1+\tfrac{(\Delta (\sigma _s)+\Delta (\theta _1)-\Delta (\theta _\infty ))(\Delta (\sigma _s)+\Delta (\theta _t)-\Delta (\theta _0))}{2\Delta (\sigma _s)} z + \sum _{k=2}^\infty c_k z^k\right) . \end{aligned}$$

The discovery of the AGT relation [2] between two-dimensional conformal field theories and four-dimensional supersymmetric gauge theories led to a closed-form expression for all the coefficients $c_k$. In our notation, the complete expansion of (3.9) can be found in [15, Eq. (3.1)]. Several conjectures exist for the analytic properties of four-point Virasoro conformal blocks. The series in (3.9) is believed to be convergent inside the unit disk $|z|<1$. Moreover, the only singularities of the conformal blocks as a function of *z* are expected to be branch points at $0,1,\infty$ [10, 42]. Under this assumption, conformal blocks are naturally defined for $z \in \mathbb {C} \setminus ((-\infty ,0 ] \cup [1,\infty ))$. Finally, they are believed to be analytic in $\varvec{\theta }$ and meromorphic in $\sigma _s$, with the only possible poles located at $\pm \sigma _s^{(m,n)}=-\frac{i}{2}(m b+n b^{-1})$ for $m,n \in \mathbb {Z}_{>0}$.

Let us finally mention that $\mathcal F$ admits another representation called Zamolodchikov’s recursion [42]. This representation is a power series of the nome

3.10 $$\begin{aligned} q(z)=\exp \left( -\pi ~ \frac{_2F_1\left( \tfrac{1}{2}, \tfrac{1}{2}, 1, 1-z\right) }{_2F_1\left( \tfrac{1}{2},\tfrac{1}{2},1,z\right) }\right) , \end{aligned}$$

where $_2F_1(a,b,c,z)$ is the hypergeometric function. Whereas the representation (3.9) is expected to converge for $|z|<1$, Zamolodchikov’s recursion converges faster than (3.9) and for all $z\in \mathbb {C} \backslash \{1\}$.

### Crossing transformations

The linear span (3.9) of four-point Virasoro conformal blocks with different internal dimensions $\sigma _s$ forms an infinite-dimensional representation of $\Gamma (\Sigma _{0,4})=\text {PSL}_2(\mathbb {Z})$, the mapping class group of the four-puncture Riemann sphere. It is generated by the braiding *B* and fusion *F* moves, such that $F^2=(BF)^3=1$. The three ways of splitting four points on $\mathbb {C}\mathbb {P}^1$ into two pairs define the *s*-, *t*- and *u*-channel bases for the space of conformal blocks. The cross-ratio argument of conformal blocks in these channels is chosen from $\{z,\frac{z}{z-1}\}$,$\{1-z,\frac{z-1}{z}\}$, and $\{\frac{1}{z},\frac{1}{1-z}\}$, respectively. The braiding move *B* acts on the *s*-channel conformal blocks as follows:

$$\begin{aligned} \mathcal F\left( b,\varvec{\theta },\sigma _s;z \right) = e^{\pm i\pi \left( \Delta (\sigma _s)-\Delta (\theta _0)-\Delta (\theta _t)\right) } \left( 1-z\right) ^{-2\Delta (\theta _t)} \mathcal F\left( b,\tilde{\varvec{\theta }},\sigma _s;\tfrac{z}{z-1} \right) ,\quad \text {Im} \,z \gtrless 0, \end{aligned}$$

where $\tilde{\varvec{\theta }}$ is obtained by performing the permutation $\theta _1 \leftrightarrow \theta _\infty$ on $\varvec{\theta }$. On the other hand, the fusion move was conjectured in [21] and established in [36]. It is represented by the integral transform

3.11 $$\begin{aligned} \qquad \mathcal F\left( b,\varvec{\theta },\sigma _s;z \right) = \int _{\mathbb R_+}d\sigma _t ~ F\left( b,\varvec{\theta },\sigma _s,\sigma _t \right) \mathcal F\left( b,\hat{\varvec{\theta }},\sigma _t;1-z \right) , \end{aligned}$$

where $\hat{\varvec{\theta }}$ is obtained by performing the permutation $\theta _0 \leftrightarrow \theta _1$ on $\varvec{\theta }$. It will be convenient to explicitly write $\hat{\varvec{\theta }}$ as

3.12 $$\begin{aligned} \hat{\varvec{\theta }} = K \varvec{\theta }, \qquad K=\begin{pmatrix} 0 &{} 0 &{} 1 &{} 0 \\ 0 &{} 1 &{} 0 &{} 0 \\ 1 &{} 0 &{} 0 &{} 0 \\ 0 &{} 0 &{} 0 &{} 1 \end{pmatrix}. \end{aligned}$$

Finally, the kernel $F\left( b,\varvec{\theta },\sigma _s,\sigma _t \right)$ of the fusion move is the Virasoro fusion kernel.

## The Virasoro fusion kernel

In this section, we recall the Ponsot–Teschner formula [21] for the Virasoro fusion kernel. Let us also note that other representations for *F* were found in [38]. Moreover, we describe various symmetry and eigenfunction properties of *F* which will be useful in its identification with the Ruijsenaars hypergeometric function (2.3).

### Definition

The Virasoro fusion kernel is defined by a contour integral involving two special functions $s_b(x)=g_b(x)/g_b(-x)$ and $g_b(x)$ which are closely related to the hyperbolic gamma function $G(a_-,a_+,x)=E(a_-,a_+,x)/E(a_-,a_+,-x)$ and $E(a_-,a_+,x)$, respectively. The exact relations are given in (A.9). The Ponsot–Teschner formula reads

4.1 $$\begin{aligned} \begin{aligned} F\left( b,\varvec{\theta },\sigma _s,\sigma _t \right) =&\prod _{\epsilon ,\epsilon '=\pm } \frac{g_b \left( \epsilon \theta _1+\theta _{t}+\epsilon ' \sigma _t\right) g_b \left( \epsilon \theta _0-\theta _\infty +\epsilon ' \sigma _t \right) }{g_b \left( \epsilon \theta _0 + \theta _t + \epsilon ' \sigma _s \right) g_b \left( \epsilon \theta _1-\theta _\infty +\epsilon ' \sigma _s \right) } \prod _{\epsilon =\pm } \frac{g_b(\frac{iQ}{2}+2\epsilon \sigma _s)}{g_b(-\frac{iQ}{2}+2\epsilon \sigma _t)} \\&\times \int _{\mathsf {F}} {\mathrm {d}}x~\prod _{\epsilon =\pm 1} \frac{s_b \left( x+ \epsilon \theta _1 \right) s_b \left( x+\epsilon \theta _0+\theta _\infty +\theta _t \right) }{s_b \left( x+\frac{iQ}{2}+\theta _\infty +\epsilon \sigma _s \right) s_b \left( x+\frac{i Q}{2}+\theta _t+\epsilon \sigma _t \right) }. \end{aligned} \end{aligned}$$

When $b>0$, the integrand in (4.1) has eight vertical semi-infinite lines of poles, four of them increasing and the other four decreasing; the integration contour $\mathsf {F}$ runs from $-\infty$ to $+\infty$, separating the increasing and decreasing sequences of poles. More generally, the fusion kernel (4.1) can be extended to a meromorphic function of all of its parameters provided that $c \in \mathbb {C} \setminus \mathbb {R}_{\le 1}$, which corresponds to $b\notin i \mathbb {R}$.

### Symmetry properties

We now present various symmetry properties of the Virasoro fusion kernel. First, because the Virasoro conformal block (3.9) is a function of the conformal dimensions $\Delta (x)=\frac{Q^2}{4}+x^2$, it follows that it is even in $\varvec{\theta }$ and $\sigma _s$. Therefore, the fusion transformation (3.11) implies that the Virasoro fusion kernel $F\left( b,\varvec{\theta },\sigma _s,\sigma _t \right)$ is even in $\varvec{\theta }$ and $\sigma _s,\sigma _t$. Second, because $s_b(x)=s_{b^{-1}}(x)$ and $g_b(x)=g_{b^{-1}}(x)$, *F* has the following symmetry:

4.2 $$\begin{aligned} F\left( b,\varvec{\theta },\sigma _s,\sigma _t \right) = F\left( b^{-1},\varvec{\theta },\sigma _s,\sigma _t \right) . \end{aligned}$$

We now describe a duality transformation exchanging the internal momenta $\sigma _s$ and $\sigma _t$.

#### Proposition 4.1

The Virasoro fusion kernel given in (4.1) satisfies

4.3 $$\begin{aligned} F\left( b,\varvec{\theta },\sigma _s,\sigma _t \right) = \frac{\alpha (\varvec{\theta },\sigma _s)\alpha (\varvec{\theta },-\sigma _s)}{\alpha (\hat{\varvec{\theta }},\sigma _t)\alpha (\hat{\varvec{\theta }},-\sigma _t)} F\left( b,\hat{\varvec{\theta }},\sigma _t,\sigma _s \right) , \end{aligned}$$

where $\hat{\varvec{\theta }}$ is related to $\varvec{\theta }$ as in (3.12) and

4.4 $$\begin{aligned} \alpha (\varvec{\theta },\sigma _s)=\frac{g_b\left( 2\sigma _s+\frac{iQ}{2} \right) g_b\left( 2\sigma _s-\frac{iQ}{2} \right) }{\prod _{\epsilon ,\epsilon '=\pm } g_b(\sigma _s+\epsilon \theta _1 +\epsilon '\theta _\infty ) g_b(\sigma _s+\epsilon \theta _0+\epsilon '\theta _t)}. \end{aligned}$$

#### Proof

The proof relies on the fact that the contour integral in (4.1) is a hyperbolic Barnes integral $\mathcal {B}_h$ (see (B.2) for its definition). In particular, $\mathcal {B}_h$ satisfies the remarkable identity (B.4) which is crucial for the proof.

Following the definition (B.2) and the relation $s_b(z)=G(b,b^{-1},z)$, it is easy to verify that the contour integral in (4.1) takes the form

4.5 $$\begin{aligned} \begin{aligned} \int _{\mathsf {F}} {\mathrm {d}}x~\prod _{\epsilon =\pm } \frac{s_b \left( x+ \epsilon \theta _1 \right) s_b \left( x+\epsilon \theta _0+\theta _\infty +\theta _t \right) }{s_b \left( x+\frac{iQ}{2}+\theta _\infty +\epsilon \sigma _s \right) s_b \left( x+\frac{i Q}{2}+\theta _t+\epsilon \sigma _t \right) } = \frac{1}{2} \mathcal {B}_h(b,b^{-1},\varvec{u}), \end{aligned} \end{aligned}$$

where $\varvec{u} \in \mathcal {G}_{iQ}$ and $\mathcal {G}_k$ is defined by (B.1). Various choices for the eight parameters $\varvec{u}$ lead to the same contour integral. Here, we choose

4.6 $$\begin{aligned} \begin{aligned}&u_1 = \tfrac{iQ}{2}+\theta _\infty +\sigma _s, \quad u_2 = \tfrac{iQ}{2}+\theta _\infty -\sigma _s, \quad u_3=\theta _1, \quad u_4=-\theta _1, \\&u_5=-\theta _0-\theta _\infty -\theta _t, \quad u_6=\theta _0-\theta _\infty -\theta _t, \quad u_7 = \tfrac{iQ}{2}+\theta _t-\sigma _t, \quad u_8=\tfrac{iQ}{2}+\theta _t+\sigma _t. \end{aligned}\end{aligned}$$

We are now going to apply the identity (B.4) satisfied by the hyperbolic Barnes integral. First, from (B.3) the action of $\omega$ on $\varvec{u} \in \mathcal {G}_{iQ}$ is given by

4.7 $$\begin{aligned} \begin{aligned}&(\omega \varvec{u})_1=\tfrac{iQ}{2}+\sigma _s, \quad (\omega \varvec{u})_2 = \tfrac{iQ}{2}-\sigma _s, \quad (\omega \varvec{u})_3 = \theta _1-\theta _\infty , \quad (\omega \varvec{u})_4=-\theta _1-\theta _\infty , \\&(\omega \varvec{u})_5 = -\theta _0-\theta _t, \quad (\omega \varvec{u})_6=\theta _0-\theta _t, \quad (\omega \varvec{u})_7 = \tfrac{iQ}{2}+\theta _\infty +\theta _t-\sigma _t, \quad (\omega \varvec{u})_8=\tfrac{iQ}{2}+\theta _\infty +\theta _t+\sigma _t. \end{aligned}\end{aligned}$$

Second, a straightforward application of the identity (B.4) yields

4.8 $$\begin{aligned} \mathcal {B}_h(b,b^{-1},\varvec{u}) = \mathcal {B}_h(b,b^{-1},\omega \varvec{u}) \prod _{\epsilon ,\epsilon '=\pm } s_b(\epsilon \sigma _s+\epsilon ' \theta _1-\theta _\infty ) s_b(\epsilon \sigma _t+\epsilon '\theta _0+\theta _\infty ). \end{aligned}$$

Recalling (4.5) and using $s_b(z)=g_b(z)/g_b(-z)$, substitution of (4.8) into (4.1) leads to

4.9 $$\begin{aligned} F\left( b,\varvec{\theta },\sigma _s,\sigma _t \right) = X_1~\mathcal {B}_h(b,b^{-1},\omega \varvec{u}), \end{aligned}$$

where

4.10 $$\begin{aligned} X_1 = \frac{1}{2} \prod _{\epsilon ,\epsilon '=\pm } \frac{g_b \left( \epsilon \theta _1+\theta _{t}+\epsilon ' \sigma _t\right) g_b \left( \epsilon \theta _0+\theta _\infty +\epsilon ' \sigma _t \right) }{g_b \left( \epsilon \theta _0 + \theta _t + \epsilon ' \sigma _s \right) g_b \left( \epsilon \theta _1+\theta _\infty +\epsilon ' \sigma _s \right) } \prod _{\epsilon =\pm } \frac{g_b(\frac{iQ}{2}+2\epsilon \sigma _s)}{g_b( -\frac{iQ}{2}+2\epsilon \sigma _t)}. \end{aligned}$$

On the other hand, using the representation (4.1) and taking $x \rightarrow x+\theta _t$, it can be verified that we also have

4.11 $$\begin{aligned} F\left( b,I\hat{\varvec{\theta }},\sigma _t,\sigma _s \right) = X_2~\mathcal {B}_h(b,b^{-1},\omega \varvec{u}), \end{aligned}$$

where $\hat{\varvec{\theta }}$ is given in (3.12), $I=\text {diag}(1,-1,1,1)$, and

4.12 $$\begin{aligned} X_2 = \frac{1}{2} \prod _{\epsilon ,\epsilon '=\pm } \frac{g_b \left( \epsilon \theta _0-\theta _{t}+\epsilon ' \sigma _s\right) g_b \left( \epsilon \theta _1-\theta _\infty +\epsilon ' \sigma _s \right) }{g_b \left( \epsilon \theta _1 - \theta _t + \epsilon ' \sigma _t \right) g_b \left( \epsilon \theta _0-\theta _\infty +\epsilon ' \sigma _t \right) } \prod _{\epsilon =\pm } \frac{g_b(\frac{iQ}{2}+2\epsilon \sigma _t)}{g_b( -\frac{iQ}{2}+2\epsilon \sigma _s)}. \end{aligned}$$

Since the Virasoro fusion kernel is even in the external momenta, we have $F\left( b,I\hat{\varvec{\theta }},\sigma _t,\sigma _s \right) =F\left( b,\hat{\varvec{\theta }},\sigma _t,\sigma _s \right)$. We deduce from (4.9) and (4.11) that the following identity holds:

4.13 $$\begin{aligned} F\left( b,\varvec{\theta },\sigma _s,\sigma _t \right) = \frac{X_1}{X_2}~F\left( b,I\hat{\varvec{\theta }},\sigma _t,\sigma _s \right) , \end{aligned}$$

and a direct computation shows that

4.14 $$\begin{aligned} \frac{X_1}{X_2} = \frac{\alpha (\varvec{\theta },\sigma _s)\alpha (\varvec{\theta },-\sigma _s)}{\alpha (\hat{\varvec{\theta }},\sigma _t)\alpha (\hat{\varvec{\theta }},-\sigma _t)}, \end{aligned}$$

where $\alpha$ is given in (4.4). Hence, the identity (4.3) is obtained. $\square$

### Difference equations

We now show that the Virasoro fusion kernel is a joint eigenfunction of four difference operators, the first two acting on the internal momentum $\sigma _s$ and the remaining two acting on its dual $\sigma _t$.

#### First pair of difference equations

Define the difference operator $H_F\left( b,\varvec{\theta },\sigma _s\right)$ by

4.15 $$\begin{aligned} H_F\left( b,\varvec{\theta },\sigma _s\right) := H_F^+\left( b,\varvec{\theta },\sigma _s\right) e^{ib\partial _{\sigma _s}}+H_F^+\left( b,\varvec{\theta },-\sigma _s\right) e^{-ib\partial _{\sigma _s}}+H_F^0\left( b,\varvec{\theta },\sigma _s\right) , \end{aligned}$$

where

4.16 $$\begin{aligned} H_F^+\left( b,\varvec{\theta },\sigma _s\right) =4\pi ^2 \frac{\Gamma \left( 1+2b^2-2ib\sigma _s \right) \Gamma \left( b^2-2i b \sigma _s \right) \Gamma \left( -2i b \sigma _s \right) \Gamma \left( 1+b^2-2ib \sigma _s \right) }{\prod _{\epsilon ,\epsilon '=\pm 1}\Gamma \left( \tfrac{bQ}{2}-ib(\sigma _s+\epsilon \theta _1+\epsilon ' \theta _\infty )\right) \Gamma \left( \tfrac{bQ}{2}-ib (\sigma _s+\epsilon \theta _0+\epsilon ' \theta _t )\right) },\end{aligned}$$

and

4.17 $$\begin{aligned} \begin{aligned}&H_F^0\left( b,\varvec{\theta },\sigma _s\right) =-2{\text {cosh}}{\left( 2\pi b \left( \theta _1+\theta _t+\tfrac{ib}{2}\right) \right) }\\&\quad +4 \displaystyle \sum _{k=\pm } \frac{\prod _{\epsilon =\pm } {\text {cosh}}{\left( \pi b\left( \epsilon \theta _\infty -\tfrac{ib}{2}-\theta _1-k\sigma _s\right) \right) } {\text {cosh}}{\left( \pi b\left( \epsilon \theta _0-\tfrac{ib}{2}-\theta _t-k\sigma _s\right) \right) }}{{\text {sinh}}{\left( 2\pi b\left( k\sigma _s+\frac{ib}{2}\right) \right) }{\text {sinh}}{\left( 2\pi b k \sigma _s \right) }}. \end{aligned} \end{aligned}$$

In the CFT literature, the operator $H_F$ represents the action of a *Verlinde loop operator* denoted $L_{\gamma _t}$ on the s-channel conformal blocks (3.8). Such an operator is obtained by computing a delicate quantum monodromy operation on the s-channel blocks (3.8) along a certain closed curve $\gamma _t$ on the four-point Riemann sphere. The computation leading to $H_F$ was explicitly realized in [1, Eq. (5.37)]. It was also performed in [8, Eq. (5.31)] in a different normalization for the conformal blocks.

We now present a direct proof of the fact that the Virasoro fusion kernel (4.1) is an eigenfunction of $H_F$. In order to provide rigorous proofs, in the remainder of this article we will need the following assumption on the parameters:

##### Assumption 4.2

*(Restriction on the parameters)* We assume that

4.18 $$\begin{aligned} 0< b < 1,\quad \varvec{\theta } \in \mathbb {R}^4, \quad (\sigma _s,\sigma _t) \in \mathbb {R}^2. \end{aligned}$$

Assumption 4.2 implies that the integration contour $\mathsf {F}$ in the Virasoro fusion kernel (4.1) is any curve going from $-\infty$ to $+\infty$ lying in the strip $\text {Im} \,x\in ]-\tfrac{Q}{2},0[$. Moreover, Assumption 4.2 is made primarily for simplicity; we expect all the results of this article to admit an analytic continuation to more general values of the parameters, such as $b\in \mathbb {C}\backslash i\mathbb {R},~ {\varvec{\theta }} \in \mathbb {C}^4$ and $(\sigma _s,\sigma _t) \in \mathbb {C}^2$.

##### Proposition 4.3

The Virasoro fusion kernel satisfies the following pair of difference equations:

4.19a $$\begin{aligned}&H_F\left( b,{\varvec{\theta }},\sigma _s\right) F\left( b,{\varvec{\theta }},\sigma _s,\sigma _t\right) = 2{\text {cosh}}{\left( 2\pi b \sigma _t\right) } ~ F\left( b,{\varvec{\theta }},\sigma _s,\sigma _t\right) , \end{aligned}$$

4.19b $$\begin{aligned}&H_F\left( b^{-1},{\varvec{\theta }},\sigma _s\right) F\left( b,{\varvec{\theta }},\sigma _s,\sigma _t\right) = 2{\text {cosh}}{\left( 2\pi b^{-1} \sigma _t\right) } ~ F\left( b,{\varvec{\theta }},\sigma _s,\sigma _t\right) . \end{aligned}$$

Although the pair of difference Eq. (4.19) is known, we are not aware of a direct proof of it. More precisely, Eq. (4.19) are special cases of the pentagon equation which is one relation of the Moore–Seiberg groupoid [16, 17, 21]. They also arise from the fact that the fusion transformation (3.11) diagonalizes the Verlinde loop operator $L_{\gamma _t}$ [37]. We now present a direct proof of (4.19).^2^

##### Proof

We only need to prove (4.19a) thanks to the symmetry (4.2) of *F*. It will be convenient to rewrite the definition (4.1) of *F* as follows:

4.20 $$\begin{aligned} F\left( b,{\varvec{\theta }},\sigma _s,\sigma _t\right) = \int _{\mathsf {F}} {\mathrm {d}}x ~ X_F(x,\sigma _s) Y_F(x,\sigma _t) Z_F(x), \end{aligned}$$

where the dependence of $X_F(x,\sigma _t), Y_F(x,\sigma _s)$ and $Z_F(x)$ on *b* and ${\varvec{\theta }}$ is omitted for simplicity, and

4.21 $$\begin{aligned} \begin{aligned}&X_F(x,\sigma _s)=\prod _{\epsilon =\pm } \frac{g_b \left( \tfrac{iQ}{2}+2\epsilon \sigma _s\right) }{s_b \left( x+\frac{iQ}{2}+\theta _\infty +\epsilon \sigma _s \right) } \prod _{\epsilon ,\epsilon '=\pm } g_b \left( \epsilon \theta _0 + \theta _t + \epsilon ' \sigma _s \right) ^{-1} g_b \left( \epsilon \theta _1-\theta _\infty +\epsilon ' \sigma _s \right) ^{-1}, \\&Y_F(x,\sigma _t)=\frac{\prod _{\epsilon ,\epsilon '=\pm }g_b \left( \epsilon \theta _1+\theta _{t}+\epsilon ' \sigma _t\right) g_b \left( \epsilon \theta _0-\theta _\infty +\epsilon ' \sigma _t \right) }{\prod _{\epsilon =\pm }g_b(-\frac{iQ}{2}+2\epsilon \sigma _t)s_b \left( x+\frac{i Q}{2}+\theta _t+\epsilon \sigma _t \right) }, \\&Z_F(x)=\prod _{\epsilon =\pm } s_b \left( x+ \epsilon \theta _1 \right) s_b \left( x+\epsilon \theta _0+\theta _\infty +\theta _t \right) .\end{aligned}\end{aligned}$$

The proof relies on the following non-trivial identity for the action of the difference operator (4.15) on the block $X_F$:

4.22 $$\begin{aligned} \frac{H_F\left( b,{\varvec{\theta }},\sigma _s\right) X_F(x,\sigma _s)}{X_F(x,\sigma _s)}=\psi (x,\sigma _s) + 2\cosh {(2 \pi b(x+\theta _t))}, \end{aligned}$$

where

4.23 $$\begin{aligned} \psi (x,\sigma _s)=-4\prod _{\epsilon =\pm } \frac{\cosh \left( \pi b \left( x+\frac{i b}{2}+\epsilon \theta _1\right) \right) \cosh \left( \pi b \left( x+\frac{i b}{2}+\theta _\infty +\theta _t+\epsilon \theta _0 \right) \right) }{\sinh (\pi b(x+ib+\theta _\infty +\epsilon \sigma _s))}. \end{aligned}$$

Let us prove the identity (4.22). Recalling that the translation operators $e^{\pm ib\partial _{\sigma _s}}$ act on $X_F(x,\sigma _s)$ as $e^{\pm ib\partial _{\sigma _s}} X_F(x,\sigma _s)=X_F(x,\sigma _s\pm ib)$, the left-hand side of (4.22) takes the form

4.24 $$\begin{aligned} \frac{H_F\left( b,{\varvec{\theta }},\sigma _s\right) X_F(x,\sigma _s)}{X_F(x,\sigma _s)} = H_F^0\left( b,{\varvec{\theta }},\sigma _s\right) + H_F^+\left( b,{\varvec{\theta }},\sigma _s\right) \frac{X_F(x,\sigma _s+ib)}{X_F(x,\sigma _s)} + H_F^+\left( b,{\varvec{\theta }},-\sigma _s\right) \frac{X_F(x,\sigma _s-ib)}{X_F(x,\sigma _s)}. \end{aligned}$$

Using the identities (A.10) and (A.11) satisfied by the functions $g_b$ and $s_b$ together with the reflection equation $\Gamma (1-z)\Gamma (z)=\pi /{\text {sin}}{(\pi z)}$ of the (Euler) Gamma function, we obtain

4.25 $$\begin{aligned} \frac{H_F\left( b,{\varvec{\theta }},\sigma _s\right) X_F(x,\sigma _s)}{X_F(x,\sigma _s)} = H_F^0\left( b,{\varvec{\theta }},\sigma _s\right) + \phi (x,\sigma _s) + \phi (x,-\sigma _s), \end{aligned}$$

where $H_F^0$ is given in (4.17) and

4.26 $$\begin{aligned} \begin{aligned}&\phi (x,\sigma _s)\\&=-\frac{4 \sinh (\pi b (x+\theta _\infty -\sigma _s))\prod _{\epsilon =\pm } \cosh (\pi b (\epsilon \theta _0+\tfrac{i b}{2}-\theta _t+\sigma _s)) \cosh (\pi b(\epsilon \theta _1+\tfrac{i b}{2}+\theta _\infty +\sigma _s))}{\sinh (2\pi b (\sigma _s+\tfrac{i b}{2})) \sinh (2 \pi b \sigma _s) \sinh (\pi b (x+i b+\theta _\infty +\sigma _s))} . \end{aligned} \end{aligned}$$

Hence, the proof of (4.22) is equivalent to proving the identity $f_1(\sigma _s)=f_2(\sigma _s)$ where

4.27 $$\begin{aligned} \begin{aligned}&f_1(\sigma _s) = \psi (x,\sigma _s) + 2 \cosh {(2 \pi b(x+\theta _t))}, \\&f_2(\sigma _s) = H_F^0\left( b,{\varvec{\theta }},\sigma _s\right) + \phi (x,\sigma _s) + \phi (x,-\sigma _s). \end{aligned}\end{aligned}$$

We proceed as follows: Both $f_1(\sigma _s)$ and $f_2(\sigma _s)$ are meromorphic functions of $\sigma _s$. It can also be easily be checked that they are even and $i b^{-1}$-periodic in $\sigma _s$. Moreover, we have the following asymptotics:

4.28 $$\begin{aligned} \begin{aligned}&\lim _{Re(\sigma _s) \rightarrow \pm \infty } H_F^0\left( b,{\varvec{\theta }},\sigma _s\right) = 0 = \lim _{Re(\sigma _s) \rightarrow \pm \infty } \psi (x,\sigma _s), \\&\lim _{Re(\sigma _s) \rightarrow \pm \infty } \phi (x,\sigma _s) = e^{\mp 2\pi b(x+\theta _t)}, \end{aligned}\end{aligned}$$

which entail the asymptotics

4.29 $$\begin{aligned} \lim _{Re(\sigma _s) \rightarrow \pm \infty } f_1(\sigma _s) = 2\cosh {(2\pi b(x+\theta _t))} = \lim _{Re(\sigma _s) \rightarrow \pm \infty } f_2(\sigma _s). \end{aligned}$$

We now show that $f_1(\sigma _s)$ and $f_2(\sigma _s)$ have equal $\sigma _s$-residues in a horizontal period strip $\text {Im} \,\sigma _s \in [0,b^{-1}]$. It can first be verified that $f_2(\sigma _s)$ has vanishing residues at $\sigma _s=\pm \tfrac{ib}{2},0$. However, it has nonzero residues at $\sigma _s=\pm (ib+\theta _\infty +x)$ which are equal to

4.30 $$\begin{aligned} \begin{aligned}&\text {Res}(f_2(\sigma _s))|_{\sigma _s=\pm (ib+\theta _\infty +x)}\\&\quad =\pm \frac{4}{\pi b} \sinh {(2 \pi b (i b+\theta _\infty +x))}^{-1} \prod _{\epsilon =\pm } \cosh (\pi b (\tfrac{i b}{2}+x+\epsilon \theta _1)) \\&\qquad \cosh (\pi b(\tfrac{i b}{2}+\theta _\infty +\theta _t+x+\epsilon \theta _0)).\end{aligned}\end{aligned}$$

On the other hand, the only residues of $f_1(\sigma _s)$ are located at $\sigma _s=\pm (ib+\theta _\infty +x)$ and straightforward computations show that they are also equal to the right-hand side of (4.30). Hence, we have shown that the function $f_1(\sigma _s)-f_2(\sigma _s)$ is bounded and holomorphic in a horizontal period strip $\text {Im} \,\sigma _s \in [0,b^{-1}]$. These properties extend to the whole complex plane by $ib^{-1}$-periodicity in $\sigma _s$. Therefore, Liouville theorem ensures that the function $f_1(\sigma _s)-f_2(\sigma _s)$ is constant everywhere. By evaluation at $\text {Re}(\sigma _s)=+\infty$ using (4.29), we deduce that $f_1(\sigma _s)=f_2(\sigma _s)$. Hence, the identity (4.22) is proved.

We now let the difference operator $H_F$ defined in (4.15) act on the Virasoro fusion kernel (4.20). Using (4.22), we have

4.31 $$\begin{aligned} \begin{aligned} H_F\left( b,{\varvec{\theta }},\sigma _s\right) F\left( b,{\varvec{\theta }},\sigma _s,\sigma _t\right)&= \int _{\mathsf {F}} {\mathrm {d}}x~X_F(x,\sigma _s) Y_F(x,\sigma _t) Z_F(x) \psi (x,\sigma _s) \\&\quad +2 \int _{\mathsf {F}} {\mathrm {d}}x~X_F(x,\sigma _s) Y_F(x,\sigma _t) Z_F(x)\cosh {(2\pi b(x+\theta _t))}. \end{aligned} \end{aligned}$$

The next step of the proof utilizes the following identity:

4.32 $$\begin{aligned} \frac{Y_F(x-ib,\sigma _t)}{Y_F(x,\sigma _t)} = 2 \cosh (2 \pi b \sigma _t)-2 \cosh {(2\pi b(x+\theta _t))}.\end{aligned}$$

We now perform a contour shift together with $x\rightarrow x-ib$ in the first line of (4.31), and we use (4.32). Assumption 4.2 ensures that the contour does not cross any pole. We obtain

4.33 $$\begin{aligned} \begin{aligned}&H_F\left( b,{\varvec{\theta }},\sigma _s\right) F\left( b,{\varvec{\theta }},\sigma _s,\sigma _t\right) = 2\cosh {(2\pi b \sigma _t)} \int _{\mathsf {F}} {\mathrm {d}}x~X_F(x-ib,\sigma _s) Y_F(x,\sigma _t) \\&\quad Z_F(x-ib) \psi (x-ib,\sigma _s) \\&\quad - 2 \int _{\mathsf {F}} {\mathrm {d}}x~X_F(x-ib,\sigma _s) Y_F(x,\sigma _t) Z_F(x-ib) \psi (x-ib,\sigma _s)\cosh {(2 \pi b(x+\theta _t))} \\&\quad + 2 \int _{\mathsf {F}} {\mathrm {d}}x~X_F(x,\sigma _s) Y_F(x,\sigma _t) Z_F(x) \cosh {(2 \pi b(x+\theta _t))}. \end{aligned}\end{aligned}$$

Finally, the identity

4.34 $$\begin{aligned} \psi (x-ib,\sigma _s) X_F(x-ib,\sigma _s)Z_F(x-ib)=X_F(x,\sigma _s)Z_F(x) \end{aligned}$$

implies that the last two lines in (4.33) cancel, and that the first line in (4.33) yields the desired result. $\square$

#### Second pair of difference equations

The duality transformation (4.3) exchanging the internal momenta $\sigma _s$ and $\sigma _t$ implies that the Virasoro fusion kernel (4.1) satisfies another pair of difference equations where the difference operators act on $\sigma _t$. We introduce the dual operator $\tilde{H}_F$ by

4.35 $$\begin{aligned} \tilde{H}_F\left( b,\hat{{\varvec{\theta }}},\sigma _t\right) := \tilde{H}_F^+\left( b,\hat{{\varvec{\theta }}},\sigma _t\right) e^{ib\partial _{\sigma _t}}+\tilde{H}_F^+\left( b,\hat{{\varvec{\theta }}},-\sigma _t\right) e^{-ib\partial _{\sigma _t}}+H_F^0\left( b,\hat{{\varvec{\theta }}},\sigma _t\right) , \end{aligned}$$

where $H_F^0$ is given in (4.17), $\hat{{\varvec{\theta }}}$ is defined by (3.12) and

4.36 $$\begin{aligned} \begin{aligned} \tilde{H}_F^+\left( b,\hat{{\varvec{\theta }}},\sigma _t\right) = \frac{4\pi ^2~\Gamma \left( 1-b^2+2 i b\sigma _t \right) \Gamma (1+2ib\sigma _t) \Gamma \left( 2 i b \sigma _t-2 b^2\right) \Gamma \left( 2 i b \sigma _t-b^2\right) }{\prod _{\epsilon ,\epsilon '=\pm } \Gamma \left( \frac{1-b^2}{2}+ib \left( \sigma _t+\epsilon \theta _0+\epsilon ' \theta _\infty \right) \right) \Gamma \left( \frac{1-b^2}{2}+ib \left( \sigma _t+\epsilon \theta _1+\epsilon ' \theta _t\right) \right) }. \end{aligned}\end{aligned}$$

We next show that the Virasoro fusion kernel is also an eigenfunction of this difference operator.

##### Proposition 4.4

The Virasoro fusion kernel satisfies the dual pair of difference equations

4.37a $$\begin{aligned}&\tilde{H}_F\left( b,\hat{{\varvec{\theta }}},\sigma _t\right) F\left( b,{\varvec{\theta }},\sigma _s,\sigma _t\right) = 2{\text {cosh}}{\left( 2\pi b \sigma _s\right) } ~ F\left( b,{\varvec{\theta }},\sigma _s,\sigma _t\right) , \end{aligned}$$

4.37b $$\begin{aligned}&\tilde{H}_F\left( b^{-1},\hat{{\varvec{\theta }}},\sigma _t\right) F\left( b,{\varvec{\theta }},\sigma _s,\sigma _t\right) = 2{\text {cosh}}{\left( 2\pi b^{-1} \sigma _s\right) } ~ F\left( b,{\varvec{\theta }},\sigma _s,\sigma _t\right) . \end{aligned}$$

##### Proof

Again, we only need to prove (4.37a) thanks to the symmetry (4.2) of *F*. Let us first apply the duality transformation ${\varvec{\theta }} \rightarrow \hat{{\varvec{\theta }}}$ and $\sigma _s \leftrightarrow \sigma _t$ to Eq. (4.19a):

4.38 $$\begin{aligned} H_F\left( b,\hat{{\varvec{\theta }}},\sigma _t\right) F\left( b,\hat{{\varvec{\theta }}},\sigma _t,\sigma _s\right) = 2{\text {cosh}}{\left( 2\pi b \sigma _s\right) } ~ F\left( b,\hat{{\varvec{\theta }}},\sigma _t,\sigma _s\right) ,\end{aligned}$$

where $H_F$ is given in (4.15). Substituting the identity (4.3) into (4.38), we obtain

4.39 $$\begin{aligned} \left( \alpha (\hat{{\varvec{\theta }}},\pm \sigma _t)^{-1}\circ H_F\left( b,\hat{{\varvec{\theta }}},\sigma _t\right) \circ \alpha (\hat{{\varvec{\theta }}},\pm \sigma _t)\right) F\left( b,{\varvec{\theta }},\sigma _s,\sigma _t\right) = 2{\text {cosh}}{\left( 2\pi b \sigma _s\right) } ~ F\left( b,{\varvec{\theta }},\sigma _s,\sigma _t\right) ,\end{aligned}$$

where for the sake of brevity we denoted $f(a,\pm b)=f(a,b)f(a,-b)$. The operator on the left-hand side of (4.39) takes the form

4.40 $$\begin{aligned} \begin{aligned}&\alpha (\hat{{\varvec{\theta }}},\pm \sigma _t)^{-1} \circ H_F\left( b,\hat{{\varvec{\theta }}},\sigma _t\right) \circ \alpha (\hat{{\varvec{\theta }}},\pm \sigma _t) = H_F^0\left( b,\hat{{\varvec{\theta }}},\sigma _t\right) \\&\quad +\frac{\alpha (\hat{{\varvec{\theta }}},\sigma _t+ib)}{\alpha (\hat{{\varvec{\theta }}},\sigma _t)} \frac{\alpha (\hat{{\varvec{\theta }}},-\sigma _t-ib)}{\alpha (\hat{{\varvec{\theta }}},-\sigma _t)} H_F^+\left( b,\hat{{\varvec{\theta }}},\sigma _t\right) e^{ib\partial _{\sigma _t}} \\&\quad + \frac{\alpha (\hat{{\varvec{\theta }}},\sigma _t-ib)}{\alpha (\hat{{\varvec{\theta }}},\sigma _t)} \frac{\alpha (\hat{{\varvec{\theta }}},-\sigma _t+ib)}{\alpha (\hat{{\varvec{\theta }}},-\sigma _t)} H_F^+\left( b,\hat{{\varvec{\theta }}},-\sigma _t\right) e^{-ib\partial _{\sigma _t}}. \end{aligned}\end{aligned}$$

A tedious but straightforward computation using the properties (A.10) and (A.11) of the functions $g_b$ and $s_b$ shows that for $k=\pm 1$, we have

4.41 $$\begin{aligned} \frac{\alpha (\hat{{\varvec{\theta }}},\sigma _t +k ib)}{\alpha (\hat{{\varvec{\theta }}},\sigma _t)} \frac{\alpha (\hat{{\varvec{\theta }}},-\sigma _t-k ib)}{\alpha (\hat{{\varvec{\theta }}},-\sigma _t)} H_F^+\left( b,\hat{{\varvec{\theta }}},k\sigma _t\right) = \tilde{H}_F^+\left( b,\hat{{\varvec{\theta }}},k\sigma _t\right) , \end{aligned}$$

which finally implies the identity

4.42 $$\begin{aligned} \alpha (\hat{{\varvec{\theta }}},\pm \sigma _t)^{-1}\circ H_F\left( b,\hat{{\varvec{\theta }}},\sigma _t\right) \circ \alpha (\hat{{\varvec{\theta }}},\pm \sigma _t) = \tilde{H}_F\left( b,\hat{{\varvec{\theta }}},\sigma _t\right) . \end{aligned}$$

In view of (4.39), this completes the proof. $\square$

In summary, we have shown that the Virasoro fusion kernel satisfies two pairs of difference equations given in (4.19) and (4.37).

## Main result

The renormalized Ruijsenaars hypergeometric function (2.3) and the Virasoro fusion kernel (4.1) were studied in Sects. 2 and 4, respectively. Although they appear in different contexts, the two functions resemble each other. Both of them are proportional to a hyperbolic Barnes integral (B.2) and are joint eigenfunctions of four difference operators. The main result of this section is Theorem 1. It shows that *F* and $R_\text {ren}$ are the same function up to normalization.

The key to the identification of *F* and $R_\text {ren}$ will be to compare and match the four difference equations that they satisfy. Let us first compare the difference operators $H_F$ defined by (4.15) and $H_{RvD}$ given in (2.9). $H_F$ and $H_{RvD}$ appear in the left-hand sides of the difference Eqs. (4.19a) and (2.12a) satisfied by *F* and $R_\text {ren}$, respectively. Of particular importance are the “potentials” $H_F^0$ defined by (4.17) and *V* given in (2.11), because they remain invariant under any change of normalization. The first step in the identification of *F* and $R_\text {ren}$ is to show that $H_F^0$ and *V* are equal under a certain parameter correspondence.

### Ruijsenaars/CFT parameter correspondence

We now provide a parameter correspondence between the Virasoro fusion kernel $F\left( b,{\varvec{\theta }},\sigma _s,\sigma _t\right)$ and the Ruijsenaars hypergeometric function $R_\text {ren}(a_-,a_+,\varvec{\gamma },v,\hat{v})$. First of all, we have

5.1 $$\begin{aligned} b=a_-, \qquad b^{-1}=a_+. \end{aligned}$$

The second relation between the pairs $(v,\hat{v})$ and $(\sigma _s,\sigma _t)$ is simply given by

5.2 $$\begin{aligned} v=\sigma _s, \qquad \hat{v}=\sigma _t. \end{aligned}$$

Moreover, the couplings $\varvec{\gamma }$ defined by (2.1) and the external momenta ${\varvec{\theta }}$ given in (3.7) are related by

5.3 $$\begin{aligned} \varvec{\gamma }=L {\varvec{\theta }},\end{aligned}$$

where the matrix *L* reads

5.4 $$\begin{aligned} L = \begin{pmatrix}-i &{} -i &{} 0 &{} 0 \\ 0 &{} 0 &{} -i &{} i \\ i &{} -i &{} 0 &{} 0 \\ 0 &{} 0 &{} -i &{} -i \end{pmatrix}. \end{aligned}$$

Finally, the dual sets of parameters $\hat{\varvec{\gamma }}$ and $\hat{\varvec{\theta }}$, respectively, given in (2.1) and (3.12) can be related as follows. It is straightforward to verify that the matrices *J* defined by (2.2), K given in (3.12) and *L* satisfy

5.5 $$\begin{aligned} JL=LK, \end{aligned}$$

which implies

5.6 $$\begin{aligned} \hat{\varvec{\gamma }}=L \hat{{\varvec{\theta }}}.\end{aligned}$$

We emphasize that the parameter correspondence described above can be rescaled thanks to the property (2.4) of the function $R_\text {ren}$.

It can now be verified that the potentials $H_F^0$ defined by (4.17) and *V* given in (2.11) are equal:

5.7 $$\begin{aligned} V(b,b^{-1},L{\varvec{\theta }},\sigma _s) = H_F^0\left( b,{\varvec{\theta }},\sigma _s\right) . \end{aligned}$$

This identification suggests that the difference operator $H_{RvD}$ defined in (2.9) can be obtained from $H_F$ given in (4.15) after a proper change of normalization. It also suggests that the difference Eq. (4.19a) satisfied by *F* can be mapped to the difference Eq. (2.12a) satisfied by $R_\text {ren}$.

### Renormalized fusion kernel $F_{\text {ren}}$

#### Some requirements

The second step in the identification of *F* and $R_\text {ren}$ is to find a renormalized version $F_{\text {ren}}$ of *F* which satisfies a difference equation of the form (2.12a), but also three other equations of the form (2.12b), (2.12c), (2.12d). This requirement can be reformulated as follows. The difference Eq. (2.12a) satisfied by the function $R_\text {ren}$ implies the three remaining Eqs. (2.12b), (2.12c) and (2.12d) because $R_\text {ren}$ satisfies the identities (2.5) and (2.6). Moreover, using the parameter correspondence of Sect. 5.1, the self-duality (2.6) of $R_\text {ren}$ becomes

5.8 $$\begin{aligned} R_{\text {ren}}(b,b^{-1},L\varvec{\theta },\sigma _s,\sigma _t)=R_{\text {ren}}(b,b^{-1},L\hat{\varvec{\theta }},\sigma _t,\sigma _s). \end{aligned}$$

This equation can be compared to the property (4.3) of the Virasoro fusion kernel: The function $R_\text {ren}$ is self-dual, while *F* is not. We deduce that the key to the identification of *F* and $R_\text {ren}$ is to find a renormalized version $F_\text {ren}$ of *F* which (i) satisfies a difference equation of the type (2.12a), (ii) is invariant under $b\rightarrow b^{-1}$, and (iii) is self-dual.

#### Definition of $F_{\text {ren}}$

We now present a renormalized version $F_{\text {ren}}$ of *F* which satisfies the three properties (i)–(iii). First, we define renormalized conformal blocks $\mathcal {F}_\text {ren}$ by

5.9 $$\begin{aligned} \mathcal F_\text {ren}\left( b,{\varvec{\theta }},\sigma _s;z\right) = N(\theta _\infty ,\theta _1,\sigma _s) N(\sigma _s,\theta _t,\theta _0) \mathcal F\left( b,{\varvec{\theta }},\sigma _s;z\right) , \end{aligned}$$

where $\mathcal F$ is given in (3.8), and where the normalization factor *N* is

5.10 $$\begin{aligned} N(\theta _3,\theta _2,\theta _1)=\frac{g_b(-\theta _1-\theta _2-\theta _3) g_b(\theta _1-\theta _2-\theta _3) g_b(-\theta _1-\theta _2+\theta _3) g_b(\theta _1-\theta _2+\theta _3)}{g_b(-2 \theta _1+\frac{i Q}{2}) g_b(-2 \theta _2+\frac{i Q}{2}) g_b(2 \theta _3+\frac{i Q}{2})}. \end{aligned}$$

We are now going to rewrite the fusion transformation (3.11) in terms of $\mathcal {F}_\text {ren}$. We first introduce a weight function^3^

5.11 $$\begin{aligned} \omega \left( b,{\varvec{\theta }},\sigma _s\right) =\mu \left( b,{\varvec{\theta }},\sigma _s\right) \mu \left( b,{\varvec{\theta }},-\sigma _s\right) , \end{aligned}$$

where

5.12 $$\begin{aligned} \mu \left( b,{\varvec{\theta }},\sigma _s\right) =\frac{s_b(\tfrac{iQ}{2}+2\sigma _s)}{\prod _{\epsilon =\pm 1}s_b \left( \sigma _s-\theta _1+\epsilon \theta _\infty \right) s_b \left( \sigma _s-\theta _t+\epsilon \theta _0\right) }. \end{aligned}$$

The fusion transformation (3.11) can now be rewritten as

5.13 $$\begin{aligned} \qquad \mathcal F_\text {ren} \left( b,{\varvec{\theta }},\sigma _s;z\right) =\int _{\mathbb R_+} d\sigma _t ~ \omega \left( b,\hat{{\varvec{\theta }}},\sigma _t\right) ~ F_{\text {ren}}\left( b,{\varvec{\theta }},\sigma _s,\sigma _t\right) \mathcal F_\text {ren}\left( b,\hat{{\varvec{\theta }}},\sigma _t;1-z\right) , \end{aligned}$$

where the renormalized Virasoro fusion kernel $F_{\text {ren}}$ is defined by

5.14 $$\begin{aligned} F_{\text {ren}}\left( b,{\varvec{\theta }},\sigma _s,\sigma _t\right) = \omega \left( b,\hat{{\varvec{\theta }}},\sigma _t\right) ^{-1} \frac{N(\theta _\infty ,\theta _1,\sigma _s) N(\sigma _s,\theta _t,\theta _0)}{N(\theta _\infty ,\theta _0,\sigma _t) N(\sigma _t,\theta _t,\theta _1)} ~ F\left( b,{\varvec{\theta }},\sigma _s,\sigma _t\right) , \end{aligned}$$

and where *F* is defined in (4.1). In the next three propositions, we show that $F_{\text {ren}}$ satisfies the desired three properties (i)–(iii).

##### Proposition 5.1

The following identity holds:

5.15 $$\begin{aligned} F_{\text {ren}}\left( b^{-1},{\varvec{\theta }},\sigma _s,\sigma _t\right) = F_{\text {ren}}\left( b,{\varvec{\theta }},\sigma _s,\sigma _t\right) . \end{aligned}$$

##### Proof

Equation (5.15) follows from the symmetries $s_b(x)=s_{b^{-1}}(x)$ and $g_b(x)=g_{b^{-1}}(x)$. $\square$

We now prove that $F_{\text {ren}}$ is self-dual.

##### Proposition 5.2

The renormalized Virasoro fusion kernel $F_{\text {ren}}$ satisfies

5.16 $$\begin{aligned} F_{\text {ren}}\left( b,{\varvec{\theta }},\sigma _s,\sigma _t\right) = F_{\text {ren}}\left( b,\hat{{\varvec{\theta }}},\sigma _t,\sigma _s\right) . \end{aligned}$$

##### Proof

The proof consists of writing an explicit relation between $F_{\text {ren}}\left( b,{\varvec{\theta }},\sigma _s,\sigma _t\right)$ and $F_{\text {ren}}\left( b,\hat{{\varvec{\theta }}},\sigma _t,\sigma _s\right)$ using the definition (5.14) and the identity (4.3). First, from (5.14) we have

5.17 $$\begin{aligned} F_{\text {ren}}\left( b,\hat{{\varvec{\theta }}},\sigma _t,\sigma _s\right) = \omega \left( b,{\varvec{\theta }},\sigma _s\right) ^{-1} \frac{N(\theta _\infty ,\theta _0,\sigma _t) N(\sigma _t,\theta _t,\theta _1)}{N(\theta _\infty ,\theta _1,\sigma _s) N(\sigma _s,\theta _t,\theta _0)} ~F\left( b,\hat{{\varvec{\theta }}},\sigma _t,\sigma _s\right) . \end{aligned}$$

Substituting the identity (4.3) into (5.17), we have

5.18 $$\begin{aligned} F_{\text {ren}}\left( b,\hat{{\varvec{\theta }}},\sigma _t,\sigma _s\right) = \omega \left( b,{\varvec{\theta }},\sigma _s\right) ^{-1} \frac{N(\theta _\infty ,\theta _0,\sigma _t) N(\sigma _t,\theta _t,\theta _1)}{N(\theta _\infty ,\theta _1,\sigma _s) N(\sigma _s,\theta _t,\theta _0)} ~ \frac{\alpha (\hat{{\varvec{\theta }}},\sigma _t)\alpha (\hat{{\varvec{\theta }}},-\sigma _t)}{\alpha ({\varvec{\theta }},\sigma _s)\alpha ({\varvec{\theta }},-\sigma _s)} F\left( b,{\varvec{\theta }},\sigma _s,\sigma _t\right) , \end{aligned}$$

where $\alpha$ is given in (4.4). Using once again the definition (5.14), we obtain

5.19 $$\begin{aligned} \begin{aligned}&F_{\text {ren}}\left( b,\hat{{\varvec{\theta }}},\sigma _t,\sigma _s\right) = \omega \left( b,{\varvec{\theta }},\sigma _s\right) ^{-1} \frac{N(\theta _\infty ,\theta _0,\sigma _t) N(\sigma _t,\theta _t,\theta _1)}{N(\theta _\infty ,\theta _1,\sigma _s) N(\sigma _s,\theta _t,\theta _0)} \\&\quad \times \frac{\alpha (\hat{{\varvec{\theta }}},\sigma _t)\alpha (\hat{{\varvec{\theta }}},-\sigma _t)}{\alpha ({\varvec{\theta }},\sigma _s)\alpha ({\varvec{\theta }},-\sigma _s)} ~ \omega \left( b,\hat{{\varvec{\theta }}},\sigma _t\right) \frac{N(\theta _\infty ,\theta _0,\sigma _t) N(\sigma _t,\theta _t,\theta _1)}{N(\theta _\infty ,\theta _1,\sigma _s) N(\sigma _s,\theta _t,\theta _0)} ~ F_{\text {ren}}\left( b,{\varvec{\theta }},\sigma _s,\sigma _t\right) . \end{aligned} \end{aligned}$$

Finally, a direct computation using $s_b(x)=g_b(x)/g_b(-x)$ shows that all the prefactors in the right-hand side of (5.19) cancel out. Therefore, the identity (5.16) is obtained. $\square$

### Difference equations for $F_{\text {ren}}$

We now show that the renormalized Virasoro fusion kernel and the renormalized Ruijsenaars hypergeometric function satisfy the same four difference equations.

#### Proposition 5.3

The renormalized fusion kernel $F_{\text {ren}}$ defined by (5.14) satisfies the following difference equations:

5.20a $$\begin{aligned}&H_{RvD}(b,b^{-1},L{\varvec{\theta }},\sigma _s) F_{\text {ren}}\left( b,{\varvec{\theta }},\sigma _s,\sigma _t\right) = 2{\text {cosh}}{(2\pi b \sigma _t)}~F_{\text {ren}}\left( b,{\varvec{\theta }},\sigma _s,\sigma _t\right) , \end{aligned}$$

5.20b $$\begin{aligned}&H_{RvD}(b^{-1},b,L{\varvec{\theta }},\sigma _s) F_{\text {ren}}\left( b,{\varvec{\theta }},\sigma _s,\sigma _t\right) = 2{\text {cosh}}{(2\pi b^{-1} \sigma _t)}~F_{\text {ren}}\left( b,{\varvec{\theta }},\sigma _s,\sigma _t\right) , \end{aligned}$$

5.20c $$\begin{aligned}&H_{RvD}(b,b^{-1},L\hat{{\varvec{\theta }}},\sigma _t) F_{\text {ren}}\left( b,{\varvec{\theta }},\sigma _s,\sigma _t\right) = 2{\text {cosh}}{(2\pi b \sigma _s)}~F_{\text {ren}}\left( b,{\varvec{\theta }},\sigma _s,\sigma _t\right) , \end{aligned}$$

5.20d $$\begin{aligned}&H_{RvD}(b^{-1},b,L\hat{{\varvec{\theta }}},\sigma _t) F_{\text {ren}}\left( b,{\varvec{\theta }},\sigma _s,\sigma _t\right) = 2{\text {cosh}}{(2\pi b^{-1} \sigma _s)}~F_{\text {ren}}\left( b,{\varvec{\theta }},\sigma _s,\sigma _t\right) , \end{aligned}$$

where the matrix *L* is given in (5.4) and $H_{RvD}$ is defined by (2.9).

#### Proof

We only need to prove (5.20a) thanks to the symmetries (5.15) and (5.16) of $F_\text {ren}$. Moreover, it will be convenient to rewrite (5.14) as $F=(P(\sigma _t)/Q(\sigma _s))F_{\text {ren}}$, where

5.21 $$\begin{aligned} \begin{aligned}&P(\sigma _t) = \omega \left( b,\hat{{\varvec{\theta }}},\sigma _t\right) N(\theta _\infty ,\theta _0,\sigma _t) N(\sigma _t,\theta _t,\theta _1), \\&Q(\sigma _s) = N(\theta _\infty ,\theta _1,\sigma _s) N(\sigma _s,\theta _t,\theta _0). \end{aligned} \end{aligned}$$

Substituting $F=(P(\sigma _t)/Q(\sigma _s))F_{\text {ren}}$ into the difference Eq. (4.19a) satisfied by *F*, we obtain

5.22 $$\begin{aligned} \left( \frac{Q(\sigma _s)}{P(\sigma _t)}\circ H_F\left( b,{\varvec{\theta }},\sigma _s\right) \circ \frac{P(\sigma _t)}{Q(\sigma _s)} \right) F_{\text {ren}}\left( b,{\varvec{\theta }},\sigma _s,\sigma _t\right) = 2{\text {cosh}}{\left( 2\pi b \sigma _t\right) } ~ F_{\text {ren}}\left( b,{\varvec{\theta }},\sigma _s,\sigma _t\right) . \end{aligned}$$

The factors $P(\sigma _t)$ in the left-hand side of (5.22) cancel out, since the difference operator $H_F\left( b,{\varvec{\theta }},\sigma _s\right)$ acts on the variable $\sigma _s$. Therefore, the proof of (5.20a) consists of verifying the following identity:

5.23 $$\begin{aligned} Q(\sigma _s)\circ H_F\left( b,{\varvec{\theta }},\sigma _s\right) \circ Q(\sigma _s)^{-1} = H_{RvD}(b,b^{-1},L{\varvec{\theta }},\sigma _s), \end{aligned}$$

where $H_F$ and $H_{RvD}$ are, respectively, defined by (4.15) and (2.9). We have

5.24 $$\begin{aligned} \begin{aligned}&Q(\sigma _s)\circ H_F\left( b,{\varvec{\theta }},\sigma _s\right) \circ Q(\sigma _s)^{-1} \\&\quad = H_F^0\left( b,{\varvec{\theta }},\sigma _s\right) + \frac{Q(\sigma _s)}{Q(\sigma _s+ib)} H_F^+\left( b,{\varvec{\theta }},\sigma _s\right) e^{ib\partial _{\sigma _s}}+ \frac{Q(\sigma _s)}{Q(\sigma _s-ib)}H_F^+\left( b,{\varvec{\theta }},-\sigma _s\right) e^{-ib\partial _{\sigma _s}}, \end{aligned}\end{aligned}$$

where $H_F^+$ is given in (4.16). Using the identity (A.10) satisfied by the $g_b$-function, it is straightforward to verify that the following identities hold for $k=\pm 1$:

5.25 $$\begin{aligned} \frac{Q(\sigma _s)}{Q(\sigma _s+kib)} =&\; \frac{\Gamma \left( 1-b^2+2 i b k \sigma _s\right) \Gamma (1+2 ib k \sigma _s)}{\Gamma \left( 1+b^2-2 i b k \sigma _s \right) \Gamma \left( 1+2 b^2-2 i b k \sigma _s\right) } \nonumber \\&\times \prod _{\epsilon =\pm } \frac{\Gamma \left( \frac{b Q}{2}+ib \left( \epsilon \theta _0+\theta _t-k \sigma _s\right) \right) \Gamma \left( \frac{b Q}{2}+ib \left( \epsilon \theta _\infty +\theta _1-k \sigma _s\right) \right) }{\Gamma \left( 1-\frac{b Q}{2}+ib \left( \epsilon \theta _0+\theta _t+k \sigma _s\right) \right) \Gamma \left( 1-\frac{b Q}{2}+ib\left( \epsilon \theta _\infty +\theta _1+k \sigma _s\right) \right) }. \end{aligned}$$

We now substitute (5.25) into (5.24), and we use the reflection equation $\Gamma (1-z)\Gamma (z)=\pi /{\text {sin}}{(\pi z)}$. For $k=\pm 1$, we obtain

5.26 $$\begin{aligned} \frac{Q(\sigma _s)}{Q(\sigma _s+kib)} H_F^+\left( b,{\varvec{\theta }},k\sigma _s\right) = C(b,b^{-1},L\varvec{\theta },-k\sigma _s), \end{aligned}$$

where *C* is defined by (2.10). It finally remains to use the identification (5.7) of the potentials *V* and $H_F^0$ to obtain (5.23). $\square$

### $F_{\text {ren}}=R_{\text {ren}}$

We have shown that the renormalized Virasoro fusion kernel (5.14) and the renormalized Ruijsenaars hypergeometric function (2.3) satisfy the same four difference equations. Therefore, the two functions are proportional. We now present the main result of this article.

#### Theorem 1

The renormalized fusion kernel $F_{\text {ren}}$ given in (5.14) and the renormalized Ruijsenaars hypergeometric function defined by (2.3) are equal:

5.27 $$\begin{aligned} F_{\text {ren}}\left( b,{\varvec{\theta }},\sigma _s,\sigma _t\right) = R_{\text {ren}}(b,b^{-1},L{\varvec{\theta }},\sigma _s,\sigma _t), \end{aligned}$$

where the matrix *L* is given in (5.4).

#### Proof

The equality is not immediate and follows from the identity (B.4) satisfied by the hyperbolic Barnes integral. Let us first rewrite $F_{\text {ren}}$ using (5.14) and the representation (4.1). It will also be convenient to perform a shift $x\rightarrow x-\tfrac{iQ}{2}$ in the integrand. This amounts to lifting up all poles of the integrand by $\tfrac{iQ}{2}$. Because of Assumption 4.2, this shift maps the contour $\mathsf {F}$ to a contour $\mathsf {F}'$ which runs from $-\infty$ and $+\infty$ and lies in the strip $\text {Im} \,x \in ]0,\tfrac{Q}{2}[$. It is now straightforward to verify that $F_{\text {ren}}$ can be written as

5.28 $$\begin{aligned} \begin{aligned} F_{\text {ren}}\left( b,{\varvec{\theta }},\sigma _s,\sigma _t\right) =&\prod _{\epsilon _1=\pm } \left( \frac{s_b(\epsilon _1\sigma _t-\theta _0-\theta _\infty )}{s_b(\epsilon _1\sigma _s+\theta _1-\theta _\infty )}\prod _{\epsilon _2=\pm } s_b(\epsilon _1\sigma _s+\epsilon _2\theta _0-\theta _t) \right) \\&\times \int _{\mathsf {F}'} {\mathrm {d}}x ~ \prod _{\epsilon =\pm } \frac{s_b(x-\tfrac{iQ}{2}+\epsilon \theta _1)s_b(x-\tfrac{iQ}{2}+\theta _\infty +\theta _t+\epsilon \theta _0)}{s_b(x+\theta _\infty +\epsilon \sigma _s)s_b(x+\theta _t+\epsilon \sigma _t)}. \end{aligned}\end{aligned}$$

On the other hand, the function $R_\text {ren}$ defined in (2.3) satisfies

5.29 $$\begin{aligned} \begin{aligned} R_{\text {ren}}(b,&b^{-1},L{\varvec{\theta }},\sigma _s,\sigma _t) = \prod _{\epsilon =\pm 1} \frac{s_b(\epsilon \sigma _s-\theta _0-\theta _t)}{s_b(\epsilon \sigma _t+\theta _1+\theta _t)} \\&\times \int _{\mathsf {R}}dz~ \frac{1}{s_b(z+\tfrac{i Q}{2}) s_b(z+2 \theta _t+\tfrac{i Q}{2})}\prod _{\epsilon =\pm 1}\frac{s_b(z+\theta _0+\theta _t+\epsilon \sigma _s) s_b(z+\theta _1+\theta _t+\epsilon \sigma _t)}{s_b\left( z+\theta _0+\theta _1+\epsilon \theta _\infty +\theta _t+\tfrac{i Q}{2}\right) }, \end{aligned}\end{aligned}$$

where the contour $\mathsf {R}$ runs from $-\infty$ to $+\infty$ lying in the strip $\text {Im} \,z \in ]-\tfrac{Q}{2},0[$. Therefore, the proof of Theorem 1 consists of proving that the functions (5.28) and (5.29) are equal. Recalling the definition (B.2) of the hyperbolic Barnes integral and using the identity $s_b(z)=G(b,b^{-1},z)$, the contour integral in (5.28) takes the form

5.30 $$\begin{aligned} \int _{\mathsf {F}'} {\mathrm {d}}x ~ \prod _{\epsilon =\pm } \frac{s_b(x-\tfrac{iQ}{2}+\epsilon \theta _1)s_b(x-\tfrac{iQ}{2}+\theta _\infty +\theta _t+\epsilon \theta _0)}{s_b(x+\theta _\infty +\epsilon \sigma _s)s_b(x+\theta _t+\epsilon \sigma _t)} = \frac{1}{2} \mathcal {B}_h(b,b^{-1},\varvec{v}). \end{aligned}$$

Several choices of $\varvec{v}\in \mathcal {G}_{iQ}$ lead to the same contour integral. Here, we choose

5.31 $$\begin{aligned} \begin{aligned}&v_1= \theta _t-\sigma _t, \quad v_2=\theta _t+\sigma _t, \quad v_3=\tfrac{i Q}{2}+\theta _1, \quad v_4=\tfrac{i Q}{2}-\theta _0-\theta _\infty -\theta _t, \\&v_5=\tfrac{i Q}{2}-\theta _1, \quad v_6=\tfrac{i Q}{2}+\theta _0-\theta _\infty -\theta _t, \quad v_7=\theta _\infty -\sigma _s, \quad v_8=\theta _\infty +\sigma _s.\end{aligned}\end{aligned}$$

From (B.3), we have $s=\frac{\theta _0-\theta _1+\theta _\infty -\theta _t}{2}$, and the action of $\omega$ on the parameters $\varvec{v}\in \mathcal {G}_{iQ}$ yields

5.32 $$\begin{aligned} \omega \varvec{v} = (v_1+s,v_2+s,\ldots ,v_5-s,\ldots ,v_8-s). \end{aligned}$$

Finally, the equality between the functions (5.28) and (5.29) is found after applying the identity (B.4) on (5.30), performing the change of variable $x=-z-\frac{\theta _1}{2}-\frac{\theta _0}{2}-\frac{\theta _\infty }{2}-\frac{3\theta _t}{2}$ which maps the contour $\mathsf {F}'$ to $\mathsf {R}$, and using the identity $s_b(z)=s_b(-z)^{-1}$. $\square$

## Conclusion and perspectives

In this article, we have proved that the Virasoro fusion kernel is a joint eigenfunction of four difference operators. We have found a normalization of the conformal blocks for which the four difference operators are mapped to four versions of the quantum relativistic hyperbolic $BC_1$ Calogero–Moser Hamiltonian. We have consequently proved that the Virasoro fusion kernel and the Ruijsenaars hypergeometric function coincide up to normalization and are the quantum eigenfunction of this integrable system. We now mention a list of perspectives related to this work.

1. It would be interesting to understand the role played by the four-point Virasoro conformal blocks in the context of the present integrable system. In view of (5.13) and Theorem 1, the function $R_\text {ren}$ is the kernel of the fusion transformation relating the *s*- and *t*-channel renormalized conformal blocks. On the other hand, in [28] a unitary Hilbert space transform associated with the function $R_\text {ren}$ was constructed for special values of the couplings. We believe that this Hilbert space is in fact spanned by the four-point Virasoro conformal blocks.
2. The quantum eigenfunction of the $A_1$ relativistic hyperbolic CM system is a one-coupling specialization of the Ruijsenaars hypergeometric function [29]. It would be interesting to compare this limit to the transition limit from the Virasoro fusion kernel to the Virasoro modular kernel [11]. The latter describes how conformal blocks associated with the one-point torus transform under a mapping class group action [18].
3. A natural question is to find higher rank generalizations of our result. The $BC_N$, $N>1$ generalization of the function $R_\text {ren}$ has not yet been constructed. Higher rank analogs of the Virasoro fusion kernel are associated with $\mathcal {W}$-type algebras and also remain to be found. However, the framework developed in [33, 34] allows us, in principle, to construct such a generalization from a quantum group perspective.
4. What is the meaning of the classical and/or non-relativistic limits in the conformal blocks setting?
5. The *R*-function reduces to the celebrated Askey–Wilson polynomials in a limit where one of $v, \hat{v}$ is discretized [26]. What does this limit mean from the conformal blocks viewpoint?
6. The Askey–Wilson polynomials can be studied using representation theory of the double affine Hecke algebra and the Askey–Wilson algebra [14, 19]. An interesting program would be to generalize this algebraic study to the case of $R_\text {ren}$. We believe that Virasoro conformal blocks and their quantum monodromies provide the correct framework for such a study.
7. Various confluent limits of the Virasoro fusion kernel were constructed in [15]. It would be interesting to understand these limits from an integrable system point of view.
8. An interesting project motivated by the WZW *SL*(2, *R*) model would be to construct the fusion kernel of the affine Lie algebra $\hat{sl}_2$ and to find a connection to integrable systems.
9. Due to the role played by the Virasoro fusion kernel in the bootstrap approach to Liouville theory [21, 23], we hope that the present work can provide new insights on the role of integrability in two-dimensional CFTs.

## Appendix A. Special functions

Equation (2.3) expresses the renormalized Ruijsenaars hypergeometric function in terms of the hyperbolic gamma function $G(a_-,a_+,z)=E(a_-,a_+,z)/E(a_-,a_+,-z)$. Let us omit the dependence of *G* and *E* on $a_-$ and $a_+$ for simplicity. Following [26], the two functions are defined by

A.1 $$\begin{aligned} G(z)={\text {exp}}{\left[ i \int _0^\infty \frac{dy}{y} \left( \frac{{\text {sin}}{2yz}}{2{\text {sinh}}{(a_+y)}{\text {sinh}}{(a_-y)}}-\frac{z}{a_+ a_- y}\right) \right] }, \qquad |\text {Im} \,z|<a, \end{aligned}$$

and

A.2 $$\begin{aligned} E(z)={\text {exp}}\left[ \frac{1}{4} \int _0^\infty \frac{dy}{y}\left( \frac{1-e^{-2iyz}}{{\text {sinh}}{(a_+ y)} {\text {sinh}}{(a_- y)}}-\frac{2iz}{a_+a_- y} - \frac{z^2}{a_+ a_-}(e^{-2a_+y}+e^{-2a_-y})\right) \right] , \qquad \text {Im} \,z<\text {Re}~a, \end{aligned}$$

where $a=(a_++a_-)/2$. The functions *G* and *E* are obviously invariant under the exchange of $a_-$ and $a_+$. Moreover, *G* satisfies the difference equations

A.3 $$\begin{aligned} \frac{G(z+ia_-/2)}{G(z-ia_-/2)}=2{\text {cosh}}{(\pi z/a_+)}, \qquad \frac{G(z+ia_+/2)}{G(z-ia_+/2)}=2{\text {cosh}}{(\pi z/a_-)}. \end{aligned}$$

As long as the ratio $a_-/a_+$ stays in the subset $\mathbb {C} \setminus (-\infty , 0]$, the function *G* extends to a meromorphic function with poles and zeros given by

A.4 $$\begin{aligned}&z=-i(k+\tfrac{1}{2})a_--i(l+\tfrac{1}{2})a_+, \qquad k,l=0,1,2,\ldots , \qquad (\text {poles}), \end{aligned}$$

A.5 $$\begin{aligned}&z=i(k+\tfrac{1}{2})a_-+i(l+\tfrac{1}{2})a_+, \qquad k,l=0,1,2,\ldots , \qquad (\text {zeros}). \end{aligned}$$

Finally, the function *G* is scale invariant:

A.6 $$\begin{aligned} G(\lambda a_-, \lambda a_+, \lambda z)=G(a_-,a_,z), \quad \lambda > 0. \end{aligned}$$

The function *E* satisfies the difference equation

A.7 $$\begin{aligned} \frac{E(z+ia_-/2)}{E(z-ia_-/2)}=\frac{\sqrt{2\pi }}{\Gamma (\tfrac{1}{2}+\frac{iz}{a_+})} \left( \tfrac{a_-}{a_+}\right) ^{\tfrac{iz}{2a_+}}, \end{aligned}$$

and the difference equation obtained by exchanging $a_-$ and $a_+$ in (A.7). The function *E* is holomorphic as long as $a_-/a_+ \in \mathbb {C} \setminus (-\infty , 0]$. Moreover, *E* has zeros located at

A.8 $$\begin{aligned} z=i(k+\tfrac{1}{2})a_-+i(l+\tfrac{1}{2})a_+, \qquad k,l = 0,1,2,\ldots \end{aligned}$$

On the other hand, the Virasoro fusion kernel (4.1) is defined in terms of two special functions $s_b(z)$ and $g_b(z)$ such that $s_b(z)=g_b(z)/g_b(-z)$. They are related to the functions *G* and *E* as follows:

A.9 $$\begin{aligned} s_b(z) = G(b, b^{-1}; z), \qquad g_b(z) = \frac{1}{E(b, b^{-1}; -z)}. \end{aligned}$$

The properties of $s_b$ and $g_b$ can be deduced from the ones of *G* and *E*. Both $s_b$ and $g_b$ are obviously invariant under the exchange of *b* and $b^{-1}$. Most importantly, the function $g_b(z)$ satisfies the difference equations

A.10 $$\begin{aligned} \frac{g_b \left( z+\frac{ib}{2}\right) }{g_b\left( z-\frac{ib}{2}\right) }=\frac{b^{-ibz}\sqrt{2\pi }}{\Gamma \left( \frac{1}{2}-ibz \right) }, \qquad \frac{g_b \left( z+\frac{i}{2b}\right) }{g_b\left( z-\frac{i}{2b}\right) }=\frac{b^{-\frac{iz}{b}}\sqrt{2\pi }}{\Gamma \left( \frac{1}{2}- \frac{iz}{b} \right) }. \end{aligned}$$

Finally, the $s_b$ function satisfies

A.11 $$\begin{aligned} \frac{s_b(z+\frac{ib}{2})}{s_b(z-\frac{ib}{2})}=2{\text {cosh}}{\pi b z}, \qquad \frac{s_b(z+\frac{i}{2b})}{s_b(z-\frac{i}{2b})}=2{\text {cosh}}{\frac{\pi z}{b}}. \end{aligned}$$

## Appendix B. Hyperbolic Barnes integral

In this appendix, we define the hyperbolic Barnes integral $\mathcal B_h(a_-,a_+,\varvec{u})$ following [5]. We describe one of its symmetry properties which is the key to the proofs of Proposition 4.1 and Theorem 1.

Define the complex hyperplane $\mathcal {G}_k$ such that

B.1 $$\begin{aligned} \mathcal {G}_k = \{ u=(u_1,\ldots u_8) \in \mathbb {C}^8 | \sum _{j=1}^8 u_j=2k \}. \end{aligned}$$

The hyperbolic Barnes integral $\mathcal B_h(a_-,a_+,\varvec{u})$ is defined for $\varvec{u} \in \mathcal {G}_{2ia}$ and $a=\tfrac{a_-+a_+}{2}$ by

B.2 $$\begin{aligned} \mathcal B_h(a_-,a_+,\varvec{u}) = 2 \int _{\mathcal {C}} \frac{\prod _{j=3}^6 G\left( z-u_j\right) }{\prod _{j=1,2,7,8}^6 G\left( z+u_j\right) }dz, \end{aligned}$$

where the contour $\mathcal {C}$ goes from $-\infty$ and $+\infty$, passing in between the upper and lower sequences of poles of the integrand.

### Lemma B.1

[5, Proposition 4.7] Let $\omega$ be an operator acting on $\varvec{u} \in \mathcal {G}_{2ia}$ as

B.3 $$\begin{aligned} \omega \varvec{u} = (u_1+s,\ldots ,u_4+s,u_5-s,\ldots ,u_8-s), \qquad s=ia-\frac{1}{2}(u_1+u_2+u_3+u_4). \end{aligned}$$

The hyperbolic Barnes integral $B_h(a_-,a_+,\varvec{u})$, $\varvec{u} \in \mathcal {G}_{2ia}$ is invariant under permutations of $(u_1, u_2, u_7,u_8)$ and of $(u_3,u_4,u_5,u_6)$, and it satisfies^4^

B.4 $$\begin{aligned} \mathcal B_h(a_-,a_+,\varvec{u})=\mathcal B_h(a_-,a_+,\omega \varvec{u})\displaystyle \prod _{j=1,2}\prod _{k=3,4}G\left( i a-u_j-u_k\right) \displaystyle \prod _{j=5,6}\prod _{k=7,8}G\left( ia-u_j-u_k\right) . \end{aligned}$$

## Footnotes

1 The hyperbolic gamma function first appeared in [24] but was named as such in [25].

2 The proof is mostly inspired from the proof of [26, Theorem 3.1].

3 The weight function $\omega$ was introduced by Ruijsenaars in [28, Eq. (1.15)] in order to construct a unitary eigenfunction transform associated with the function $R_\text {ren}$.
